# Adherence to Medical Cannabis Among Licensed Patients in Israel

**DOI:** 10.1089/can.2015.0003

**Published:** 2016-01-01

**Authors:** Yuval Zolotov, Yehuda Baruch, Haim Reuveni, Racheli Magnezi

**Affiliations:** ^1^ Public Health and Health Systems Management Program, Department of Management, Bar Ilan University, Ramat-Gan, Israel.; ^2^ School of Public Health, University of Haifa, Haifa, Israel.; ^3^ Gertner Institute for Epidemiology and Health Policy Research, Sheba Medical Center, Ramat-Gan, Israel.; ^4^ Department of Health Systems Management, Ben-Gurion University of the Negev, Be'er Sheva, Israel.

**Keywords:** behavior, medical therapy, public policy, regulation

## Abstract

**Objectives:** To evaluate adherence among Israeli patients who are licensed to use medical cannabis and to identify factors associated with adherence to medical cannabis.

**Methods:** Ninety-five novice licensed patients were interviewed for this cross-sectional study. The questionnaire measured demographics, the perceived patient–physician relationship, and the level of patients' active involvement in their healthcare. In addition, patients were queried about adverse effect(s) and about their overall satisfaction from this medical treatment.

**Results:** Eighty percent (*n*=76) has been identified as adherent to medical cannabis use. Variables found associated with adherence were “country of origin” (immigrant status), “type of illness” (cancer vs. non-cancer), and “experiencing adverse effect(s).” Three predictors of adherence were found significant in a logistic regression model: “type of illness” (odds ratio [OR] 0.101), patient–physician relationship (OR 1.406), and level of patient activation (OR 1.132). 71.5% rated themselves being “completely satisfied” or “satisfied” from medical cannabis use.

**Conclusions:** Our findings show a relatively high adherence rate for medical cannabis, as well as relative safety and high satisfaction among licensed patients. Additionally indicated is the need to develop and implement standardized education about this evolving field—to both patients and physicians.

## Introduction

The use of medical cannabis is steadily increasing, both in Israel and globally,^1–3^ and the potential of cannabis as a therapeutic agent has been gaining both interest and acknowledgment.^4,5^ Nevertheless, although cannabis has been used for medical purposes throughout history,^6,7^ Western medicine generally does not accept it as a legitimate therapeutic treatment and social and legal barriers prevent adequate research in this field.^4,8,9^

The Israeli regulatory program of medical cannabis has changed through the years and is managed by a Medical Cannabis Unit of the Israeli Ministry of Health (MOH). Licenses are issued upon approval of a medical recommendation by a specialist, whose specialty is relevant to the medical problem for which medical cannabis is requested. The estimated number of patients is currently 22,000, and licenses are generally granted as a last resort for patients whose symptoms have not been alleviated by traditional medications and treatments.^10^ Nevertheless, preliminary data from MOH indicate that a subset of patients are in fact not using medical cannabis despite being licensed, namely not adhering to this treatment regimen.

Adherence to (or compliance with) a medication regimen has been defined by the WHO as “the extent to which patients take medications as prescribed by their healthcare providers.”^11^ Nonadherence affects patient outcomes, including quality of life, as well as adding unnecessary costs to the health system.^12^ Barriers to adherence have been largely categorized into three types: (1) barriers related specifically to the patient (e.g., economic status, lifestyle, anxiety or other emotional factors); (2) barriers associated with the healthcare provider (mainly patient–physician interactions); and (3) barriers related to the health system (e.g., regulations on price and accessibility).^13^

Accumulating scientific evidence suggests that cannabis may potentially be a promising therapy for different patient populations, including pain,^14–17^ oncology,^18,19^ HIV,^20^ and Crohn's disease.^21^ Amplified by public demand and political pressures, this has led to laws and regulations that hitherto permit the legal use of medical cannabis in 23 states in the United States and in several other countries worldwide, including Israel.^3,9^

Across the different medical cannabis regulations of healthcare systems around the world, physicians are functioning as gatekeepers who approve (or disapprove) the use of medical cannabis, either directly as in the United States and in the new regulatory system of Canada^1,22^ or by signing a document that entails further authorization by health authorities, as in Israel and in the old Canadian system.^18,23,24^ While few studies, mainly from the United States, have found physicians to be either skeptical or negative toward medical cannabis,^25–31^ a recent study has found Israeli physicians to be, in contrast, partially positive.^32^ In addition, an international poll conducted on the website of the *New England Journal of Medicine* has found vast support for medical cannabis use.^33^

The patient–physician relationship is an important factor in medical care and it affects positive treatment outcomes, including patient satisfaction, adherence to therapy, and even efficacy.^34^ Better patient–physician communication was found to have a crucial effect on adherence to medical treatments.^35^

In recent years, patients are empowered to participate in their medical care.^36–38^ The Patient Activation Measure (PAM) was developed^39^ to quantify the level of knowledge, experience, responsibility, and confidence that a patient has with regard to managing his or her health situation. PAM has been frequently used in different settings,^40^ and higher levels of this measure were found to be associated with different treatment outcomes, including better adherence.^36,40,41^

Despite both the utmost importance of adherence to medical regimens and the worldwide increase in medical cannabis use, to our knowledge no data about adherence to medical cannabis have been so far published. The study objectives were (1) to evaluate the adherence rate among patients who are licensed to use medical cannabis in Israel; and (2) to identify factors that are associated with adherence to medical cannabis. Based on previous studies, we hypothesized that the patient–physician relationship, as well as the level of patient activation, will be associated with better adherence.

## Methods

### Participants

The population of this cross-sectional study was patients aged 18 years or older who were issued a novel license to use medical cannabis between May and September 2013 (*n*=461). Two hundred and forty (52%) gave an initial consent, on their license application, to be contacted for research purposes. Of those, 95 (40%) patients participated in the study and answered the questionnaire through telephone interviews. The remaining 145 patients were not interviewed due to the following reasons: wrong contact details or disconnected phone numbers (17%, *n*=41); no answer to recurrent calls in three different days at three different times of the day (16.2%, *n*=39); deceased (11.2%, *n*=27); refused to participate (8.3%, *n*=20); or sick and unable to communicate (7.5%, *n*=18). All participants were contacted within 5–8 weeks of being initially licensed to use medical cannabis.

### Ethical statement

The institutional review board was granted to receive contact details of licensed patients who had given their authorization to be contacted for research purposes. Informed consent was obtained orally at the beginning of the interview, and all personal details were kept separately from patients' responses.

### Measures

The questionnaire included four sections and was reviewed by five physicians with extensive experience in medical cannabis for face and content validity and for overall reliability. The first section of the questionnaire included demographic information—gender, age, country of birth (immigrant status), education, and income level—as well as questions about tobacco use and the primary type of illness (for which medical cannabis license was granted).

In the second section, we used the PAM, a validated questionnaire developed by Hibbard et al.,^39^ to quantify patients' level of participation in their healthcare. PAM is scored on a theoretical 0–100 scale, which is also categorized to four levels of activation.^40^ This 13-item scale has been previously translated and validated in Hebrew,^42^ and internal consistency of this measure in the current study, calculated using the Cronbach's alpha test, was α=0.826.

In the third section, we adopted the Patient–Doctor Relationship Questionnaire (PDRQ-9)^43^ to evaluate how patients perceived the relationship between them and the physician who had provided them the medical recommendation (that is required before medical cannabis licensure). The PDRQ-9 is a valid and reliable measure that was developed out of the Helping Alliance Questionnaire.^44^ Validation of the Hebrew version was achieved by forward and backward translations made by two independent bilingual translators. Few of the questions were slightly revised to adapt the questionnaire for this study. For instance, the item “I can talk freely with my healthcare provider” was changed to “I can talk freely with my physician about medical cannabis.” Similarly, the item “My primary care provider and I agree on the nature of my medical symptoms” was revised to “My physician and I agree on the nature of my medical symptoms, for which I am licensed to use medical cannabis.” The internal consistency of this measure in the current study, calculated using the Cronbach's alpha test, was α=0.803.

The fourth section measured adherence and nonadherence to medical cannabis use by asking directly “are you currently using medical cannabis?” Nonadhering patients were asked whether they have ever used medical cannabis after being issued a license, and if so for how long and by which way of administration. Adhering patients were questioned about patterns of use (dosage and way of administration). Last, all participants who were using medical cannabis at the time of the interview, or beforehand, were asked to specify if they have ever experienced adverse effect(s) resulting from medical cannabis and were asked to subjectively rate their satisfaction from the medical effect(s) of medical cannabis use (on a 5-point scale from “completely unsatisfied” to “completely satisfied”).

### Statistical analyses

Chi-square tests of independence and Wilcoxon signed-rank tests were used to examine the association between the different independent variables and adherence. T tests were used to compare means of PAM and PDRQ between adhering and nonadhering patients. A binary logistic regression model was used to identify potential predictors of adherence. Odds ratios with 95% confidence intervals were calculated for potential predictors. All statistical analyses were performed using SPSS statistical software, version 20.

## Results

### Sample characteristics

Table 1 presents demographic variables, as well as tobacco use and type of illness, stratified to “adherent” (80%, *n*=76) and “nonadherent” (20%, *n*=19). Native Israelis were more adherent than immigrants [χ^2^(1)=3.8, *p*=0.05], who were mainly from former USSR (18.9%, *n*=18) and North Africa or Iraq (7.4%, *n*=7). In addition, cancer patients were less adherent to medical cannabis use than those with other sickness [χ^2^(2)=11.8, *p*<0.01].

**Table 1.** **Characteristics of the Study Sample (*N* = 95)**

Variable	Adherent, *N* (%)	Nonadherent, *N* (%)
Gender, χ^2^(1) = 0.412, *p* = 0.521		
Female	26 (34.2)	8 (42.1)
Male	50 (65.8)	11 (57.9)
Age, years, χ^2^(3) = 3.490, *p* = 0.322		
18–30	12 (16.4)	2 (10.5)
31–45	20 (27.4)	3 (15.8)
46–60	22 (30.1)	5 (26.3)
>60	19 (26.1)	9 (47.4)
Country of origin, χ^2^(1) = 3.817, *p* = 0.051		
Israel	54 (71.1)	9 (47.4)
Other	22 (28.9)	10 (52.7)
Education, years, χ^2^(3) = 0.251, *p* = 0.472		
<12	17 (22.4)	6 (31.6)
12	32 (42.1)	9 (47.4)
13–15	15 (19.7)	1 (5.3)
>16	12 (15.8)	3 (15.8)
Income, χ^2^(2) = 1.398, *p* = 0.497		
Above average	11 (14.5)	1 (5.3)
Average	18 (23.7)	4 (21.1)
Below average	47 (61.8)	14 (73.7)
Tobacco use, χ^2^(1) = 2.429, *p* = 0.119		
Yes	35 (46.1)	5 (26.3)
No	41 (53.9)	14 (73.7)
Type of illness, χ^2^(2) = 11.820, *p* = 0.003		
Cancer	24 (31.6)	14 (73.7)
Chronic pain	42 (55.3)	3 (15.8)
Other	10 (13.2)	2 (10.5)

The average scores of nonadhering patients to both PAM and PDRQ were significantly lower (*p*<0.001) than the scores of adhering patients (48.9±12.3 vs. 62.6±10.5 and 30.3±4.6 vs. 35.7±3.6, respectively).

Table 2 shows the logistic regression model of predicting adherence. With demographic variables controlled, three variables were found to be significantly predicting adherence: type of illness: cancer versus noncancer (*p*<0.05) and scores of both PAM (*p*<0.01) and PDRQ (*p*<0.01).

**Table 2.** **Logistic Regression Model Predicting Adherence to Medical Cannabis**

Variable	Odds ratio	95% CI	*p*
Age	1.034	0.978–1.093	0.237
Gender	5.705	0.815–39.953	0.079
Country of origin	3.365	0.396–28.604	0.266
Education	1.296	0.857–1.96	0.22
Income	3.762	0.357–39.597	0.27
Type of illness	0.101	0.012–0.823	0.032
PAM	1.132	1.03–1.244	0.01
PDRQ-9	1.406	1.112–1.777	0.004

95% CI, 95% confidence interval; PAM, Patient Activation Measure; PDRQ-9, Patient–Doctor Relationship Questionnaire.

### Patterns of use

Of the adhering patients (*n*=76), most were using medical cannabis by smoking, either as the sole way of administration (61.8%, *n*=47) or in addition to edibles (4.2%, *n*=4), vaporizer (2.6%, *n*=2), or ointment (2.6%, *n*=2). Other adhering patients used medical cannabis only with vaporizer (3.9%, *n*=3), as an ointment (15.8%, *n*=15), or as an edible (3.9%, *n*=3). The monthly dosage that patients were authorized to use was 20 g for most patients (93.4%, *n*=71) and 30 g to all others (6.6%, *n*=5).

Of the nonadhering patients (*n*=19), 63.1% (*n*=12) reported to have started using medical cannabis, but quit after 2 weeks on average (median: 10 days, range: 1–6 weeks). These patients reported to have used medical cannabis by smoking (36.8%, *n*=7), as an ointment (21%, *n*=4) or as an edible (0.5%, *n*=1).

### Adverse effect and patients' satisfaction

Of the 88 patients who have either been found adherent (*n*=76) or who have experienced medical cannabis before quitting (*n*=12), 30.7% (*n*=27) reported experiencing one adverse effect or more due to medical cannabis use. Experiencing adverse effect(s) was more common among nonadhering patients (58.3%, *n*=7) than among adhering patients (26.3%, *n*=20), and this association was found significant [χ^2^(1)=5, *p*<0.05]. The most common adverse effects were dizziness (17%, *n*=15), dehydrated mouth (11.3%, *n*=10), fatigue (6.8%, *n*=6), mild anxiety (5.6%, *n*=5), and feeling “weird” (5.6%, *n*=5).

Most patients were either “completely satisfied” (43.1%, *n*=38) or “satisfied” (28.4%, *n*=25) from the use of medical cannabis; 10 patients (11.3%) were “somewhat satisfied;” 10 (11.3%) were “not so satisfied;” and 5 (5.6%) were “completely unsatisfied.”

## Discussion

This study draws attention to adherence to medical cannabis, which has so far not been examined. The adherence rate we found is higher than adherence rates reported for other medical treatments in previous studies,^45–47^ and we suggest that this may be partially explained by the fact that licenses are only granted to patients who have gone through prior treatment regimens, thus already proving themselves as adherent patients. Other factors that could explain this relatively high adherence rate are the relative safety of medical cannabis (as demonstrated by patients' reports on adverse effects) and the overall high satisfaction that patients expressed from using medical cannabis.

While comparing the rates of adherence to medical cannabis to those of other treatments, one should note that the essence of adherence is profoundly different across different treatment regimens and different patient populations.^13^ Medical cannabis is essentially dissimilar to other medical treatments in many aspects. For instance, unlike other prescriptions, medical cannabis is usually administrated by smoking,^48^ and patients are usually not guided by physicians about the desired dosage and other use patterns.^27^

The country of birth (immigrant status) was the only demographic variable that was found associated with adherence, so that immigrants were more likely to be nonadherent, and this finding is consistent with other studies from Israel that reported higher rates of nonadherence among immigrants.^49,50^ It is plausible that language barriers and cultural gaps contributed to this lower adherence rate, as patients from different cultural backgrounds differ in their health behavior and their health beliefs,^51^ which in turn may affect adherence.

Echoing a meta-analysis that found an association between adherence and the (perceived) disease severity,^52^ we found cancer patients to be at greater risk of nonadherence than noncancer patients. While the majority of the noncancer patients were chronic pain patients, who probably have considerable experience in using opioids or other so-called narcotics, cancer patients may have less familiarity with such substances and therefore may be more wary of the stigmatization of using such a drug.

As we hypothesized, patient activation was indeed associated with adherence to medical cannabis use. This is consistent with previous studies which found that patient activation predicted better health outcomes and better health behaviors.^38,40,41^ Similarly, the patient–physician relationship was associated with adherence, which resembles findings of previous studies,^34,53–55^ and might imply a positive correlation between (perceived) patient–physician relationship and adherence.

Despite the inherent limitations of the cross-sectional study design, this study provides primary, yet valuable, data about the convoluted topic of our study. Our main limitation is the small sample size (*n*=95). However, this sample may be in fact representative, as gender and age distribution of the study population is similar to that of medical cannabis patients in Israel, as derived from official data of the Israeli MOH. In addition, adherence to medical treatments in general, and to medical cannabis specifically, is a complex phenomenon that incorporates biological factors, as well as behavioral, psychological, social, cultural, and others. Thus, our study design might have partially failed to capture its full complexity, which future studies should target with varied strategies. Last, our study is based on self-reports, which could be somewhat limited and biased. However, most of the items used in this study were validated measurements.

## Conclusion

Our findings show a relatively high adherence rate for medical cannabis, as well as relative safety and high satisfaction among licensed patients. Thus, medical cannabis seems to be a favorable treatment option, and physicians and regulators should enable access to eligible patients while finding the sensitive balance between the existing scientific evidence and patients' rights and preferences.

### Practice implications

Our findings point to the importance of patients' empowerment and activation, which can be mainly strengthened through education. Thus, it would be reasonable to consider the implementation of standardized patient education for medical cannabis use and to impose this education process as mandatory to all novice medical cannabis patients. As suggested by our findings, special care could be designed for cancer patients and immigrant patients, to minimize the risk of nonadherence. The patient–physician relationship was spotted as an important factor, and we suggest that this interaction could and should be improved. Possible ways of achieving this is by building a bona-fide relationship before the discussion about medical cannabis and by providing more education about medical cannabis to physicians and other healthcare providers.

Last, technological arrangements could be made to facilitate “real-time” data collection from licensed medical cannabis suppliers, to enable a more systematic surveillance of medical cannabis use, and specifically adherence to this treatment. With the goal of minimizing nonadherence and improving clinical care, nonadhering patients could be thus spotted and offered to join an intervention.

## Abbreviations Used

95% CI: 95% confidence interval
MOH: Ministry of Health
OR: odds ratio
PAM: Patient Activation Measure
PDRQ-9: Patient–Doctor Relationship Questionnaire

## References

[1] Fischer B, Kuganesan S, Room R “Medical Marijuana” programs–the better “third way” or sneaky side-door for cannabis control reform? Observations from Canada. Int J Drug Policy. 2015;26:15–19. doi:10.1016/j.drugpo.2014.09.007 25287942

[2] Belle-Isle L, Walsh Z, Callaway R, et al. Barriers to access for Canadians who use cannabis for therapeutic purposes. Int J Drug Policy. 2014. doi:10.1016/j.drugpo.2014.02.009 24947993

[3] Sznitman SR, Zolotov Y Cannabis for therapeutic purposes and public health and safety: a systematic and critical review. Int J Drug Policy. 2015;26:20–29 25304050 10.1016/j.drugpo.2014.09.005

[4] Bostwick JM Blurred boundaries: the therapeutics and politics of medical marijuana. Mayo Clin Proc. 2012;87:172–186 22305029 10.1016/j.mayocp.2011.10.003 PMC3538401

[5] Carter GT, Javaher SP, Nguyen MH, et al. Re-branding cannabis: the next generation of chronic pain medicine? Pain Manag. 2015;5:13–21 25537695 10.2217/pmt.14.49

[6] Zuardi AW History of cannabis as a medicine: a review. Rev Bras Psiquiatr. 2006;28:153–157 16810401 10.1590/s1516-44462006000200015

[7] Kalant H Medicinal use of cannabis – history and current status. Pain Res Manag. 2001;6:80–91 11854770 10.1155/2001/469629

[8] Philipsen N, Butler RD, Simon-Waterman C, et al. Medical marijuana: a primer on ethics, evidence, and politics. J Nurse Pract. 2014;10:633–640

[9] Rubens M Political and medical views on medical marijuana and its future. Soc Work Public Health. 2014;29:121–131 24405197 10.1080/19371918.2013.821351

[10] Sznitman SR, Lewis N Is cannabis an illicit drug or a medicine? A quantitative framing analysis of Israeli newspaper coverage. Int J Drug Policy. 2015. doi:10.1016/j.drugpo.2015.01.010 25661483

[11] Sabaté E, ed. Adherence to long-term therapies evidence for action. World Health Organization: Geneva, 2003 http://search.ebscohost.com/login.aspx?direct=true&scope=site&db=nlebk&db=nlabk&AN=114059 (accessed 226, 2014)

[12] Cutler DM, Everett W Thinking outside the pillbox—medication adherence as a priority for health care reform. N Engl J Med. 2010;362:1553–1555 20375400 10.1056/NEJMp1002305

[13] Osterberg L, Blaschke T Adherence to medication. N Engl J Med. 2005;353:487–497 16079372 10.1056/NEJMra050100

[14] Lucas P Cannabis as an adjunct to or substitute for opiates in the treatment of chronic pain. J Psychoactive Drugs. 2012;44:125–133 22880540 10.1080/02791072.2012.684624

[15] McQuay HJ More evidence cannabis can help in neuropathic pain. Can Med Assoc J. 2010;182:1494–1495 20805209 10.1503/cmaj.100799 PMC2950178

[16] Ware MA, Wang T, Shapiro S, et al. Smoked cannabis for chronic neuropathic pain: a randomized controlled trial. CMAJ. 2010;182:E694–E701 20805210 10.1503/cmaj.091414 PMC2950205

[17] Wilsey B, Marcotte T, Deutsch R, et al. Low-dose vaporized cannabis significantly improves neuropathic pain. J Pain. 2013;14:136–148 23237736 10.1016/j.jpain.2012.10.009 PMC3566631

[18] Bar-Sela G, Vorobeichik M, Drawsheh S, et al. The medical necessity for medicinal cannabis: prospective, observational study evaluating the treatment in cancer patients on supportive or palliative care. Evid Based Complement Alternat Med. 2013;2013:51039 2 10.1155/2013/510392 PMC3730175 23956774

[19] Machado Rocha FC, Stéfano SC, De Cássia Haiek R, et al. Therapeutic use of *Cannabis sativa* on chemotherapy-induced nausea and vomiting among cancer patients: systematic review and meta-analysis. Eur J Cancer Care (Engl). 2008;17:431–443 18625004 10.1111/j.1365-2354.2008.00917.x

[20] Abrams DI, Hilton JF, Leiser RJ, et al. Short-term effects of cannabinoids in patients with HIV-1 infection: a randomized, placebo-controlled clinical trial. Ann Intern Med. 2003;139:258–266 12965981 10.7326/0003-4819-139-4-200308190-00008

[21] Naftali T, Bar-Lev Schleider L, Dotan I, et al. Cannabis induces a clinical response in patients with Crohn's disease: a prospective placebo-controlled study. Clin Gastroenterol Hepatol. 2013;11:1276–1280.e1 23648372 10.1016/j.cgh.2013.04.034

[22] Aggarwal SK, Carter GT, Sullivan MD, et al. Medicinal use of cannabis in the United States: historical perspectives, current trends, and future directions. J Opioid Manag. 2009;5:153–168. http://www.oregon.gov/pharmacy/imports/marijuana/staffinfo/medicinaluseofcannabisintheus.pdf Accessed 1123, 2013 19662925 10.5055/jom.2009.0016

[23] Eggertson L New medical marijuana regulations shift onus to doctors to prescribe. Can Med Assoc J. 2013;185:E553–E554 23820445 10.1503/cmaj.109-4528 PMC3761021

[24] Shelef A, Mashiah M, Schumacher I, et al. Medical grade cannabis (MGC): regulation mechanisms, the present situation around the world and in Israel [in Hebrew]. Harefuah. 2011;150:913–917, 935, 934 22352285

[25] Charuvastra A, Friedmann PD, Stein MD Physician attitudes regarding the prescription of medical marijuana. J Addict Dis. 2005;24:87–93 16186085 10.1300/J069v24n03_07

[26] Kondrad E, Reid A Colorado family physicians' attitudes toward medical marijuana. J Am Board Fam Med. 2013;26:52–60 23288281 10.3122/jabfm.2013.01.120089

[27] Fletcher J Marijuana is not a prescription medicine. Can Med Assoc J. 2013;185:36 9 10.1503/cmaj.130267 PMC3602249 23479689

[28] Thompson JW, Koenen MA Physicians as gatekeepers in the use of medical marijuana. J Am Acad Psychiatry Law. 2011;39:460–464 22159973

[29] Wilkinson ST, D'Souza DC Problems with the medicalization of marijuana. JAMA. 2014;311:2377–2378 24845238 10.1001/jama.2014.6175 PMC6248328

[30] Saper CB Up in smoke: a neurologist's approach to “medical marijuana.” Ann Neurol. 2015;77:13–14 25472938 10.1002/ana.24327

[31] Hill KP Medical marijuana: more questions than answers. J Psychiatr Pract. 2014;20:389–391 25226202 10.1097/01.pra.0000454786.97976.96 PMC4243838

[32] Ebert T, Zolotov Y, Eliav S, et al. Assessment of Israeli physicians' knowledge, experience and attitudes towards medical cannabis: a pilot study. Isr Med Assoc J. 2015;17:437–441 26357721

[33] Adler JN, Colbert JA Medicinal use of marijuana—polling results. N Engl J Med. 2013;368:e3 0 23718175 10.1056/NEJMclde1305159

[34] Haskard Zolnierek KB, DiMatteo MR Physician communication and patient adherence to treatment: a meta-analysis. Med Care. 2009;47:826–834 19584762 10.1097/MLR.0b013e31819a5acc PMC2728700

[35] Joosten EAG, DeFuentes-Merillas L, de Weert GH, et al. Systematic review of the effects of shared decision-making on patient satisfaction, treatment adherence and health status. Psychother Psychosom. 2008;77:219–226 18418028 10.1159/000126073

[36] Bodenheimer T, Lorig K, Holman H, et al. Patient self-management of chronic disease in primary care. JAMA. 2002;288:2469–2475 12435261 10.1001/jama.288.19.2469

[37] Holman H, Lorig K Patient self-management: a key to effectiveness and efficiency in care of chronic disease. Public Health Rep. 2004;119:23 9 15158102 10.1016/j.phr.2004.04.002 PMC1497631

[38] Rask KJ, Ziemer DC, Kohler SA, et al. Patient activation is associated with healthy behaviors and ease in managing diabetes in an indigent population. Diabetes Educ. 2009;35:622–630 19419972 10.1177/0145721709335004

[39] Hibbard JH, Stockard J, Mahoney ER, et al. Development of the patient activation measure (PAM): conceptualizing and measuring activation in patients and consumers. Health Serv Res. 2004;39(4 Pt 1):1005–1026 15230939 10.1111/j.1475-6773.2004.00269.x PMC1361049

[40] Greene J, Hibbard JH Why does patient activation matter? An examination of the relationships between patient activation and health-related outcomes. J Gen Intern Med. 2012;27:520–526 22127797 10.1007/s11606-011-1931-2 PMC3326094

[41] Marshall R, Beach MC, Saha S, et al. Patient activation and improved outcomes in HIV-infected patients. J Gen Intern Med. 2013;28:668–674 23288378 10.1007/s11606-012-2307-y PMC3631066

[42] Magnezi R, Glasser S Psychometric properties of the Hebrew translation of the patient activation measure (PAM-13). Brucki S, ed. PLoS One. 2014;9:e113391 10.1371/journal.pone.0113391 PMC4239053 25411841

[43] Van der Feltz-Cornelis CM, Van Oppen P, Van Marwijk HW, et al. A patient-doctor relationship questionnaire (PDRQ-9) in primary care: development and psychometric evaluation. Gen Hosp Psychiatry. 2004;26:115–120 15038928 10.1016/j.genhosppsych.2003.08.010

[44] Luborsky L, Barber JP, Siqueland L, et al. The revised Helping Alliance questionnaire (HAq-II): psychometric properties. J Psychother Pract Res. 1996;5:26 0 22700294 PMC3330423

[45] Cramer JA A systematic review of adherence with medications for diabetes. Diabetes Care. 2004;27:1218–1224 15111553 10.2337/diacare.27.5.1218

[46] DiMatteo MR Variations in patients' adherence to medical recommendations: a quantitative review of 50 years or research. Med Care. 2004;42:200–209 15076819 10.1097/01.mlr.0000114908.90348.f9

[47] Fischer MA, Stedman MR, Lii J, et al. Primary medication non-adherence: analysis of 195,930 electronic prescriptions. J Gen Intern Med. 2010;25:284–290 20131023 10.1007/s11606-010-1253-9 PMC2842539

[48] Kalant H Smoked marijuana as medicine: not much future. Clin Pharmacol Ther. 2008;83:517–519 18349871 10.1038/sj.clpt.6100497

[49] Vinker S, Shani M, Baevsky T, et al. Adherence with statins over 8 years in a usual care setting. Am J Manag Care. 2008;14:388–392 18554077

[50] Wilf-Miron R, Peled R, Yaari E, et al. The association between socio-demographic characteristics and adherence to breast and colorectal cancer screening: analysis of large sub populations. BMC Cancer. 2011;11:37 6 21867544 10.1186/1471-2407-11-376 PMC3176246

[51] Kaplan RC, Bhalodkar NC, Brown EJ, et al. Race, ethnicity, and sociocultural characteristics predict noncompliance with lipid-lowering medications. Prev Med. 2004;39:1249–1255 15539064 10.1016/j.ypmed.2004.04.041

[52] DiMatteo MR, Haskard KB, Williams SL Health beliefs, disease severity and patient adherence: a meta-analysis. Med Care. 2007;45:521–528 17515779 10.1097/MLR.0b013e318032937e

[53] Arbuthnott A, Sharpe D The effect of physician–patient collaboration on patient adherence in non-psychiatric medicine. Patient Educ Couns. 2009;77:60–67 19395222 10.1016/j.pec.2009.03.022

[54] Butow P, Sharpe L The impact of communication on adherence in pain management. Pain. 2013 10.1016/j.pain.2013.07.048 23928026

[55] Thompson L, McCabe R The effect of clinician-patient alliance and communication on treatment adherence in mental health care: a systematic review. BMC Psychiatry. 2012;12:8 7 22828119 10.1186/1471-244X-12-87 PMC3528426

